# Supplementary material for: Genome-aware annotation of CRISPR guides validates targets in variant cell lines and enhances discovery in screens
Source: Genome Med. 2024 Nov 26;16:139. doi: 10.1186/s13073-024-01414-4 (PMC11590575; doi:10.1186/s13073-024-01414-4)
Supplement: Supplementary file 1 — Additional file 1: Figures. Supplementary analysis and benchmarking. [file 13073_2024_1414_MOESM1_ESM.docx]

**Genome-aware annotation of CRISPR guides validates targets in variant cell lines and enhances discovery in screens**

Simon Lam^1,#^, John C. Thomas^1^, Stephen P. Jackson^1,#^

^1^ Cancer Research UK Cambridge Institute, University of Cambridge, Li Ka Shing Centre, Robinson Way, Cambridge, CB2 0RE, United Kingdom.

^#^ Corresponding authors: [simon.lam@cruk.cam.ac.uk](mailto:simon.lam@cruk.cam.ac.uk) (SL); [steve.jackson@cruk.cam.ac.uk](mailto:steve.jackson@cruk.cam.ac.uk) (SPJ).

**Additional file 1**

Fig. S1.


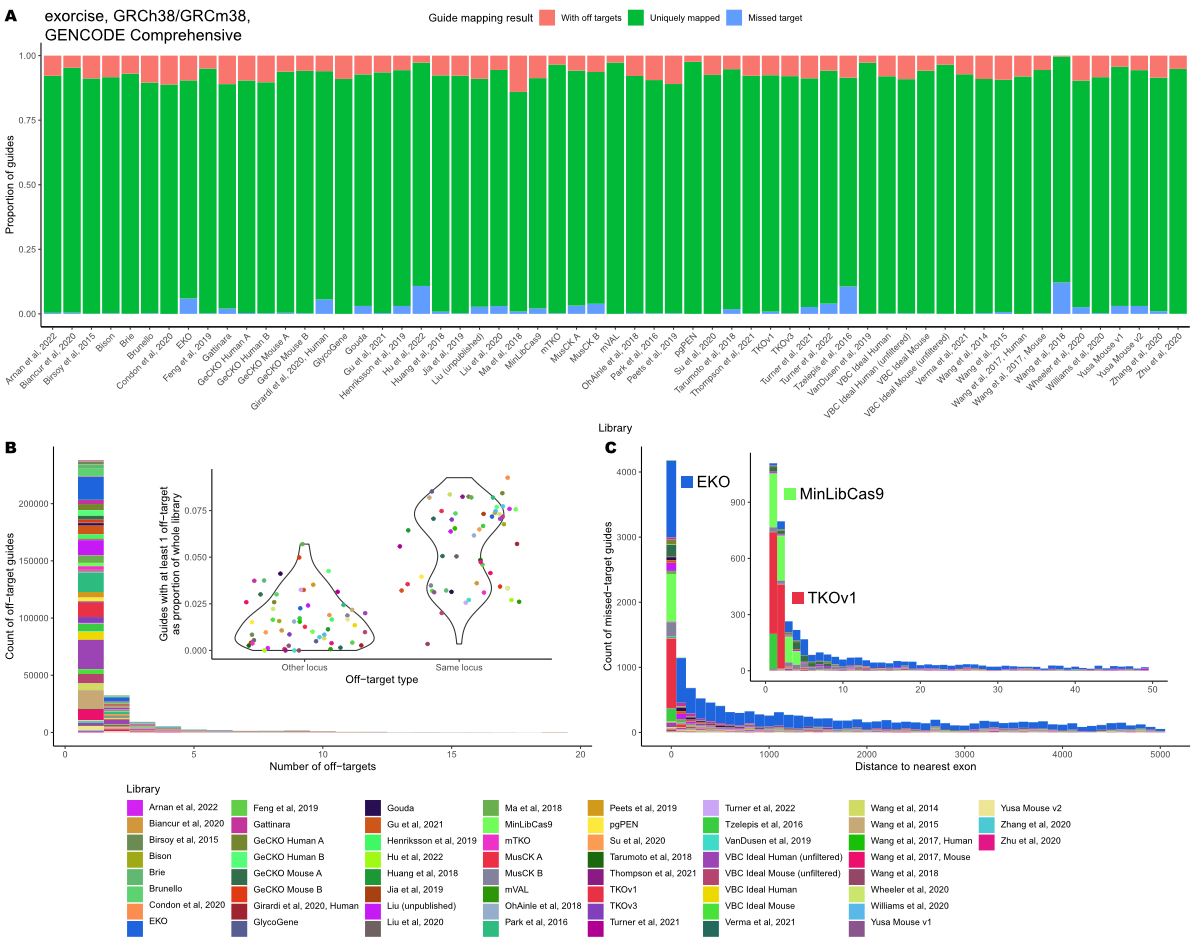


Fig. S2.


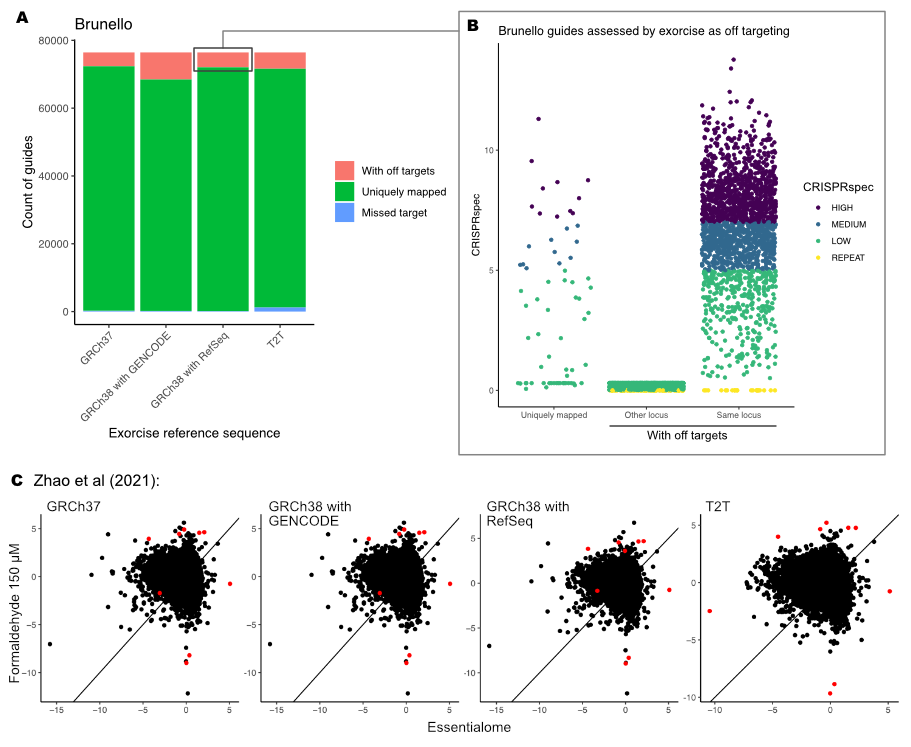


Fig. S3.


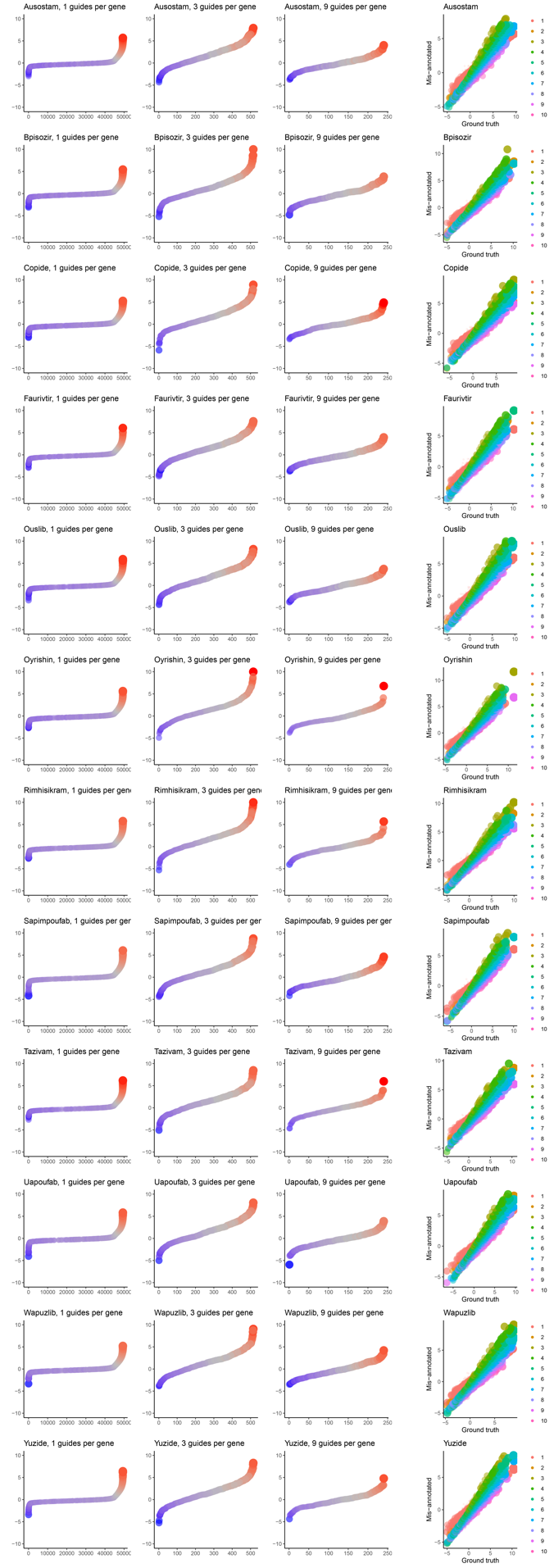


Fig. S4.


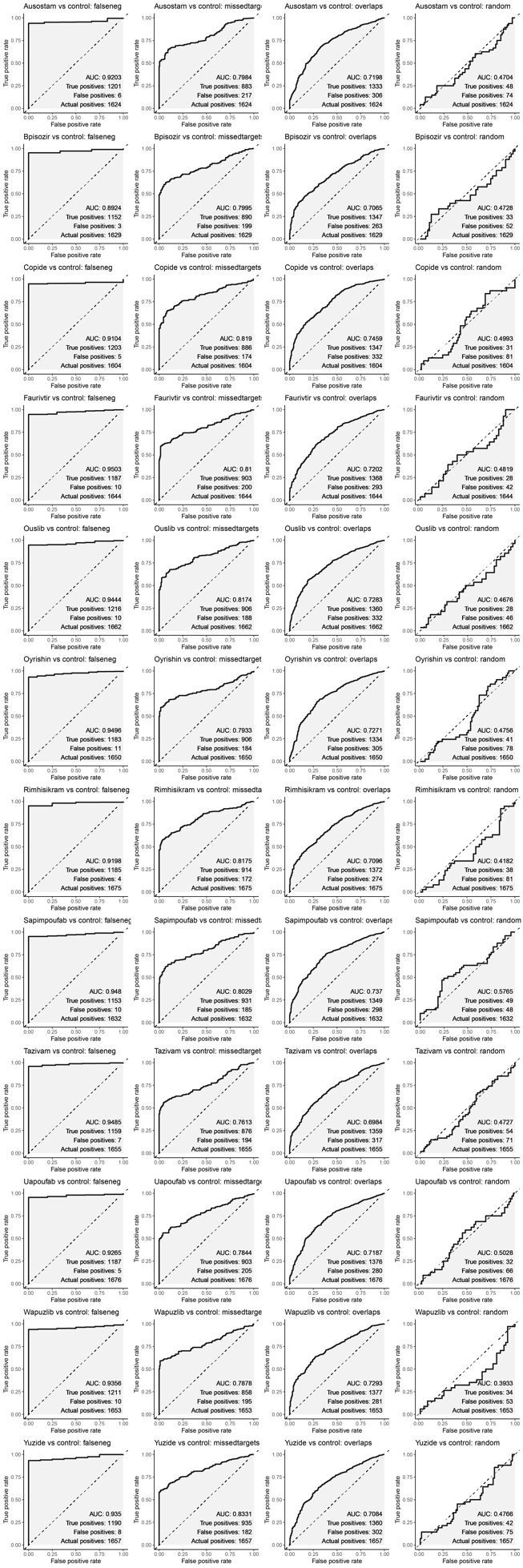


Fig. S5.


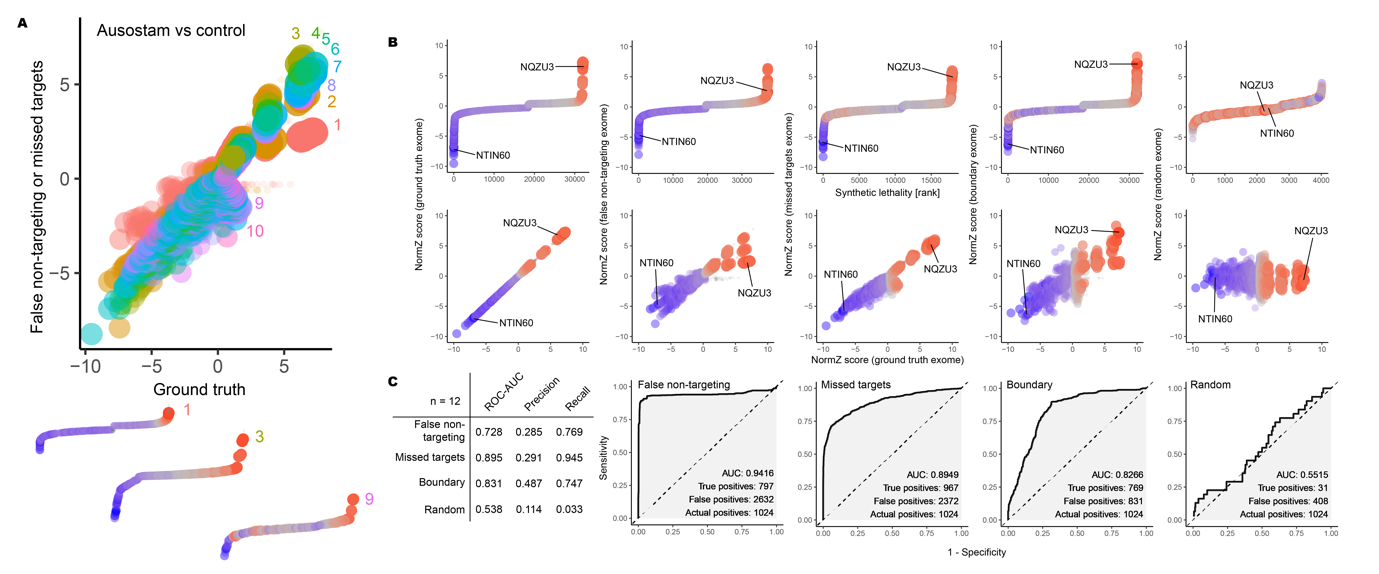


Fig. S6.


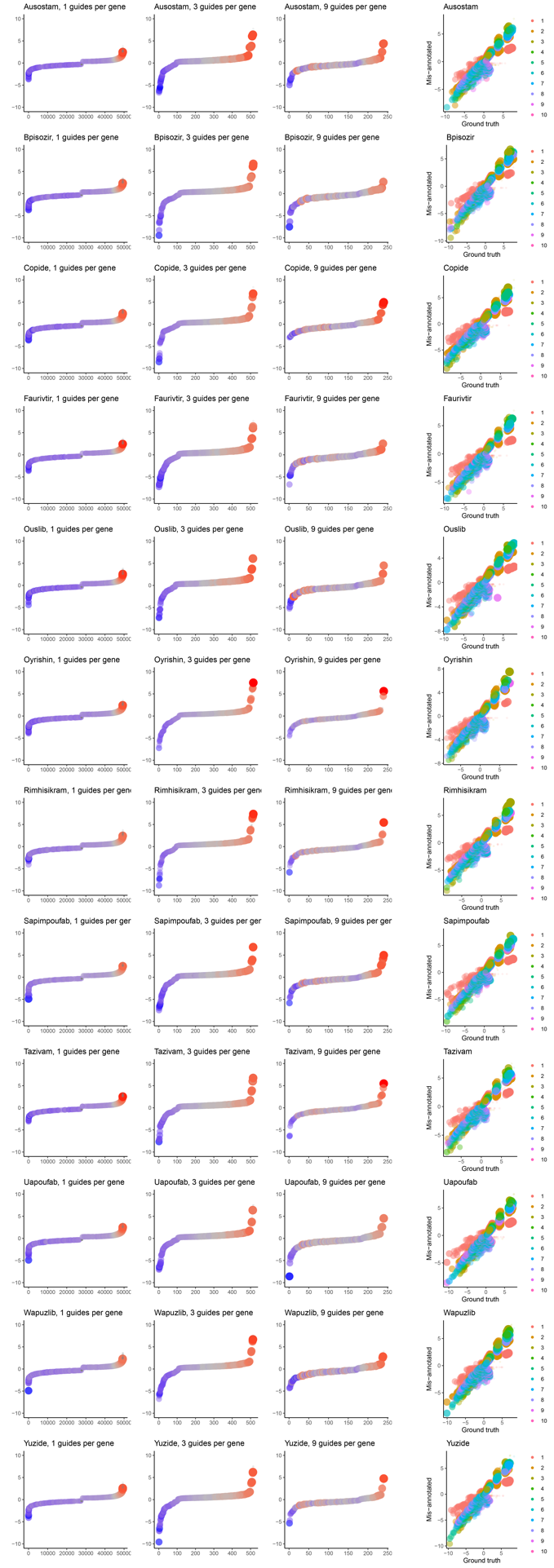


Fig. S7.


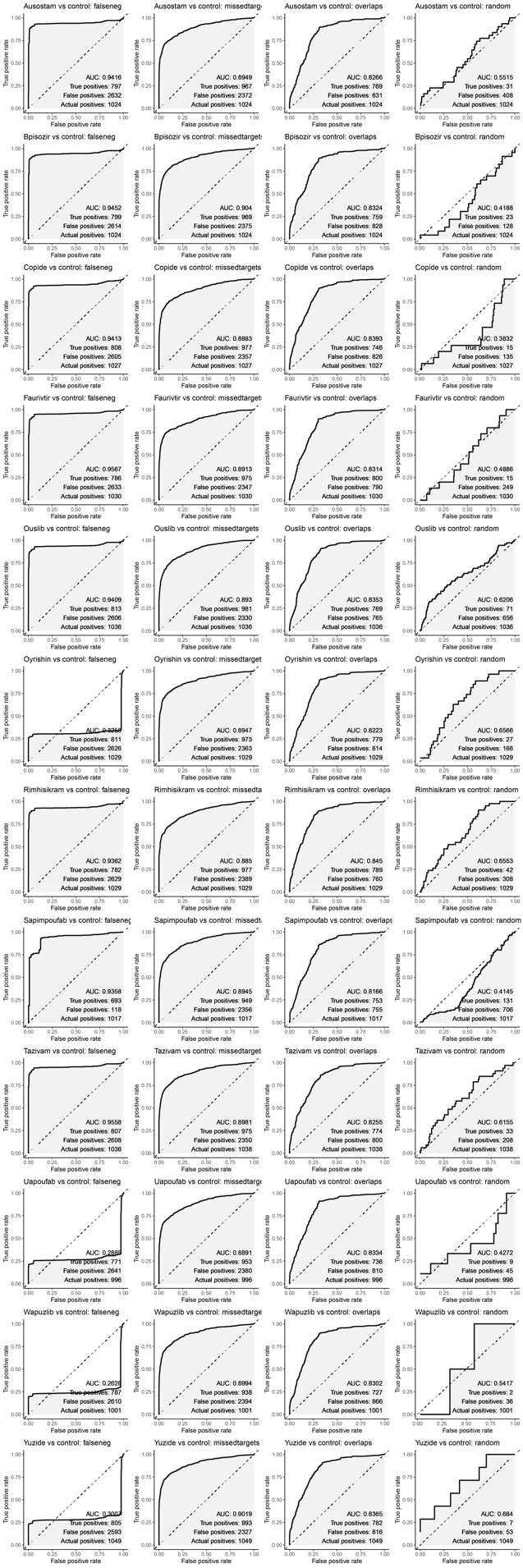


**Figure legends**

**Fig. S1.** Assessment of Addgene pooled CRISPR-spCas9 libraries for human and mouse using Exorcise with GENCODE Comprehensive. A) As in Fig. 2C. B) As in Fig. 2E. C) As in Fig. 2F.

**Fig. S2.** Brunello library benchmark. A) Exorcise re-annotation performance with different reference sequences, as in Fig. 2C. B) Comparison of Exorcise off-target identification with CRISPRoff analysis. A sample of on-target guides were included in the analysis as a reference. C) Hit-calling performance of a CRISPR screen by Zhao et al (2021)^20^ with Exorcise with different reference sequences. Red points indicate the same genes in each plot.

**Fig. S3.** Rank plots and bundle analysis for the simulated mis-annotated CRISPR screen, DrugZ analysis. Rank plots as in Fig. 3C upper. Bundle analysis as in Fig. 3B upper.

**Fig. S4.** Receiver-operator characteristic (ROC) analysis for the simulated mis-annotated CRISPR screen, DrugZ analysis. As in Fig. 3D. Falseneg, false non-targeting mis-annotation scheme; missedtargets, missed targets mis-annotation scheme; overlaps, boundary mis-annotation scheme.

**Fig. S5.** Simulated CRISPR screen on synthetic data, MAGeCK analysis. A) As in Fig. 3B. B) As in Fig. 3C. C) As in Fig. 3D.

**Fig. S6.** Rank plots and bundle analysis for the simulated mis-annotated CRISPR screen, MAGeCK analysis. Rank plots as in Additional File 1: Fig. S4B upper. Bundle analysis as in Additional File 1: Fig. S4A upper.

**Fig. S7**. Receiver-operator characteristic (ROC) analysis for the simulated mis-annotated CRISPR screen, MAGeCK analysis. As in Additional File 1: Fig. S4C except that the positive classification threshold was 0.9 rather than 0.5.

**Table S1.** Source data for Fig. 2C.

**Table S2.** Source data for Fig. 2E.

**Table S3.** Source data for Fig. 2E inset.

**Table S4.** Source data for Fig. 2F.

**Table S5.** VBC Ideal Human CRISPR-spCas9 knockout guide library after Exorcise with RefSeq.

**Table S6.** VBC Ideal Mouse CRISPR-spCas9 knockout guide library after Exorcise with RefSeq.

**Table S7.** Simulated chemo-genetic interaction values, related to Fig. 3A.

**Table S8.** Synthetic guide sequences, related to Fig. 3A.

**Table S9.** Ground truth and mis-annotated exomes, related to Fig. 3A.

**Table S10.** Simulated CRISPR screen counts, related to Fig. 3A.

**Table S11.** Source data for Fig. 3B upper.

**Table S12.** Source data for Fig. 3B lower, trace 1.

**Table S13.** Source data for Fig. 3B lower, trace 3.

**Table S14.** Source data for Fig. 3B lower, trace 9.

**Table S15.** Source data for Fig. 3C upper, panel 1.

**Table S16.** Source data for Fig. 3C upper, panel 2.

**Table S17**. Source data for Fig. 3C upper, panel 3.

**Table S18**. Source data for Fig. 3C upper, panel 4.

**Table S19.** Source data for Fig. 3C upper, panel 5.

**Table S20**. Source data for Fig. 3C lower, panel 1.

**Table S21**. Source data for Fig. 3C lower, panel 2.

**Table S22.** Source data for Fig. 3C lower, panel 3.

**Table S23.** Source data for Fig. 3C lower, panel 4.

**Table S24**. Source data for Fig. 3C lower, panel 5.

**Table S25**. Source data for Fig. 4B upper.

**Table S26**. Source data for Fig. 4B lower.

**Table S27**. Source data for Fig. 5A and 5D.

**Table S28**. Source data for Fig. 5B panel 1.

**Table S29.** Source data for Fig. 5B panel 2.

**Table S30**. Source data for Fig. 5B panel 3.

**Table S31**. Source data for Fig. 5B panel 4.

**Table S32**. Source data for Fig. 5E.

**Table S33**. Source data for Additional File 1: Fig. S1A.

**Table S34**. Source data for Additional File 1: Fig. S1B.

**Table S35.** Source data for Additional File 1: Fig. S1B inset.

**Table S36**. Source data for Additional File 1: Fig. S1C.

**Table S37**. Source data for Additional File 1: Fig. S5B upper, panel 1.

**Table S38**. Source data for Additional File 1: Fig. S5B upper, panel 2.

**Table S39**. Source data for Additional File 1: Fig. S5B upper, panel 3.

**Table S40**. Source data for Additional File 1: Fig. S5B upper, panel 4.

**Table S41**. Source data for Additional File 1: Fig. S5B upper, panel 5.

**Table S42**. Source data for Additional File 1: Fig. S5B lower, panel 1.

**Table S43.** Source data for Additional File 1: Fig. S5B lower, panel 2.

**Table S44.** Source data for Additional File 1: Fig. S5B lower, panel 3.

**Table S45**. Source data for Additional File 1: Fig. S5B lower, panel 4.

**Table S46**. Source data for Additional File 1: Fig. S5B lower, panel 5.

**Table S47.** Source data for Additional File 1: Fig. S2A.

**Table S48.** Source data for Additional File 1: Fig. S2B.

**Table S49.** Source data for Additional File 1: Fig. S2C, panel 1.

**Table S50.** Source data for Additional File 1: Fig. S2C, panel 2.

**Table S51.** Source data for Additional File 1: Fig. S2C, panel 3.

**Table S52.** Source data for Additional File 1: Fig. S2C, panel 4.
